# A Longitudinal Investigation Into the Relationship Between Working as a Speech and Language Therapist and Wellbeing

**DOI:** 10.1111/1460-6984.70046

**Published:** 2025-05-21

**Authors:** Claire Ewen, Craig A. Jackson, John Galvin

**Affiliations:** ^1^ School of Social Sciences Birmingham City University Edgbaston Birmingham UK; ^2^ School of Social Sciences Birmingham City University Bartholomew Row Birmingham UK

**Keywords:** job satisfaction, occupational stress, speech and language therapist, speech‐language pathologist, wellbeing

## Abstract

**Background:**

Healthcare workers risk stress, burnout and fatigue from time pressures, insufficient workload control and ineffective support. Unaddressed, these issues can lead to the attrition of the workforce. Retention of speech and language therapists (SLTs) is an ongoing concern, but little is known about the relationship between SLT wellbeing and their workplaces.

**Aims:**

The aims of the present study were therefore to: (1) Explore the levels of job satisfaction and general wellbeing of SLTs working clinically. (2) Describe the occupational environments of SLTs working clinically. (3) Investigate the relationship between job design and wellbeing outcomes. (4) Establish whether job satisfaction and general psychosocial wellbeing levels were consistent over time. (5) Explore whether personal changes/changes to work made by participants over a 3‐month period impacted their job satisfaction and/or general wellbeing.

**Method:**

All SLTs practising clinically in the UK were eligible to participate. Participants were recruited via selective sampling (advertising in Royal College of Speech and Language Therapists publications) and snowballing (using social media). A longitudinal study, using an online survey at two time points (P1 and P2), approximately 3 months apart, measured work stressors of SLTs, job satisfaction and general wellbeing, using the Speech‐language Pathologist Stress Inventory, Generic Job Satisfaction Survey and General Health Questionnaire‐28 (GHQ‐28). Relationships between job types, wellbeing and subsequent changes were analysed using multivariate analysis of variance.

**Results:**

Of the 632 participants at P1, 98% were women, 95% were white and the average age was 38.7 years. Ninety‐one percent were working in England, and 85% were organisationally employed. At P1, 53.3% of participants who completed the GHQ‐28 reported anxiety, depression, somatic symptoms of stress and social dysfunction. Those in high‐demand, low‐control, and low‐support (‘Isostrain’) jobs (*n* = 105) had the lowest job satisfaction (*p* < 0.001, partial *η* ^2^ = 0.383) and poorest wellbeing (*p* < 0.001, partial *η* ^2^ = 0.210). Wellbeing scores were stable from P1 to P2 (*n* = 295). Making ‘positive personal changes’ had no impact on job satisfaction, although changing jobs did (*t* (16) = −3.225, *p* < 0.01).

**Conclusion and Implications:**

Over half the participants in this study reported psychosocial ill health associated with the demands, control and support in their jobs. Psychosocial risks are embedded within work organisation, suggesting that employers and managers of SLTs consider the overall psychosocial design of jobs, with a view to improving retention. The use of a step‐by‐step risk assessment and intervention approach is recommended. Further research may corroborate the results and ensure better psychosocial risk management.

**WHAT THIS PAPER ADDS:**

*What is already known on the subject*

- The extant literature on speech and language therapist (SLT) wellbeing reports various levels of job satisfaction and burnout. While this has been associated with several job factors, there is yet to be a comprehensive analysis of how job design impacts wellbeing. This study aimed to investigate this and provide a starting point for improving wellbeing through the consideration of job design.

*What this paper adds to the existing knowledge*

- Results of this study indicate that, before the onset of COVID‐19, just over one in two SLTs working clinically in the UK were at risk of being psychologically vulnerable to anxiety, depression, experiencing somatic symptoms of stress, and social dysfunction. Those who experienced high demand, low control and low support at work were more likely to experience psychological distress and low job satisfaction.

*What are the potential or actual clinical implications of this work?*

- This study has revealed a combination of potential workplace features that are associated with SLT wellbeing, suggesting employers consider the overall psychosocial design of SLT jobs, instead of viewing occupational factors individually. The JDCS model proved a suitable framework for describing SLT jobs; it could be used in the future to gain detailed knowledge about different elements that constitute particular SLT jobs. It is essential that the voices of SLTs who are primarily clinical are heard and used to inform this appraisal; collaboration between managers and clinicians is necessary for success in this endeavour.

## 1 Introduction

Speech and language therapists (SLTs) are a valuable human resource in the workplaces in which they are employed. Fostering their wellbeing is therefore good for them, good for the organisations where they work and good for service users. According to the World Health Organization, health care workers are at risk of occupational stress, burnout and fatigue due to time pressures, a lack of control over work tasks, long working hours and a lack of support (WHO 2025). Individual efforts, such as attending yoga or owning a pet, have been associated with better wellbeing (Alexander 2015; McConnell et al. 2019). However, occupational wellbeing is the responsibility of both the employee and the employer. Employers have the ability to design favourable workplaces for health workers, including SLTs; it is therefore incumbent on them to manage the possible causes of stress identified by the WHO (2025). Further research into the relationship between working as an SLT and wellbeing is necessary. The purpose of the present study was to explore this relationship, using a longitudinal design to allow measurement of the effect that personal changes (e.g., practicing mindfulness), and/or workplace‐based changes might have on wellbeing.

### 1.1 Theoretical Models

The term ‘wellbeing’ deserves definition: In addition to the physical (biological) elements of health, the Biopsychosocial (BPS) model (Engel 1977) includes the psychological (e.g., anxiety) and social (e.g., feeling isolated) components of wellbeing. The way that work contributes to these elements of wellbeing is described in the Job Demand Control Support (JDCS) model (Johnson and Hall 1988; Karasek 1979). The model has been used extensively in studies investigating the psychosocial work experience (Häusser et al. 2010). Within the JDCS model, psychological demand is conceptualised as workload. Control is comprised of ‘decision latitude’ (autonomy) and ‘skill discretion’ (a variety of work tasks providing opportunities to utilise different skills and control over which specific skills to use to accomplish tasks). Finally, support refers to work‐related social support from both coworkers and supervisors/managers.

**FIGURE 1:**
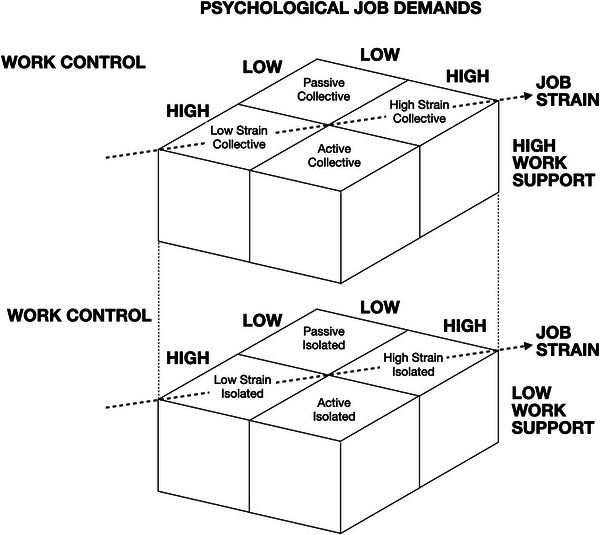
The job demand‐control‐support model (Johnson and Hall 1988).

The principal prediction of the JDCS model is that high psychological demands with low control will result in a ‘high strain’ job, associated with increased stress, decreased job satisfaction, and exhaustion and depression (Karasek 1979). High‐strain jobs are also potentially associated with physical outcomes, for example, an increased risk of cardiovascular disease due to higher blood pressure and raised serum cholesterol (Karasek and Theorell 1990). In addition to the ‘high strain’ job, Karasek (1979) proposed three more outcomes of the demand–control relationship. Firstly, he posited that combining high demand *and* high control would result in an ‘active job’, where learning and motivation are optimised, and job satisfaction is high. Secondly, jobs with low demand and low control would result in ‘passive jobs’, associated with low satisfaction, and finally, those with low demands and high control would lead to ‘low strain’ jobs. Support (or the lack thereof) serves to either buffer the impact of demands or exacerbate those demands. Jobs with good support are classified as being ‘collective’, while those with limited support are ‘isolated’. Circumstances characterised by both high strain and high isolation are labelled ‘Iso‐strain jobs’, and this job type is posited as being the most detrimental to wellbeing. The JDCS model is presented in Figure 1.

### 1.2 Health Worker Wellbeing

The WHO's (2025) statement regarding workplace risks to healthcare practitioner wellbeing is supported by research reporting the occupational and mental health of these workers. Allied health professionals have been reported to experience psychological distress due to high demands and low support (Bonsaken et al. 2021), and targeting job control can improve job satisfaction and mental health in nurses (Elliott et al. 2017). Across the limited extant literature on SLT wellbeing, insights into two areas are provided: the job satisfaction of SLTs, and the stress/burnout that they experience resulting from their work.

Research has produced mixed findings regarding levels of SLT job satisfaction. Although some studies have reported average SLT job satisfaction (Blood et al. 2002a), others have found low satisfaction for “operating conditions” (Kalkhoff and Collins 2012, p. 172), and an intention to leave the profession due to a lack of job satisfaction (Heritage et al. 2019). Factors contributing to low levels of job satisfaction have included unmanageable workloads (Smith‐Randolph and Johnson 2005). However, reporting regarding the relationship between job satisfaction and caseload size has been mixed. Blood et al. (2002a) reported a negative correlation between the two, but Kalkhoff and Collins (2012) found that caseload size did not predict satisfaction.

Although some studies have described low levels of SLT stress (Blood et al. 2002b; Blood et al. 2002c); burnout has been variably reported as being present in 99.7% (Kasbi et al. 2018) and 51% (Kaegi et al. 2002) of SLTs, depicting some variation across different settings. Stress and burnout have been associated with limited autonomy and a lack of professional support (LPS) (Bruschini, et al. 2018; Blood et al. 2002b). Previous studies, however, have not taken a holistic view of how job design and the combination/interaction of job features is linked to SLT wellbeing, possibly explaining conflicting findings around wellbeing. A systematic review of wellbeing, job satisfaction and burnout in SLTs by Ewen et al. (2021) included 17 studies and concluded that there is a need for more theoretically driven studies. Furthermore, not enough longitudinal research has been carried out.

The aims of the present study were therefore to:

1. Explore the levels of job satisfaction and of general wellbeing of SLTs working clinically.
2. Describe the occupational environments of SLTs working clinically, through the application of the JDCS model.
3. Investigate the relationship between JDCS job types and wellbeing outcomes.
4. Establish whether job satisfaction and general psychosocial wellbeing levels were consistent over time.
5. Explore whether personal changes made by participants over a 3‐month period impacted their job satisfaction and/or mental wellbeing.
6. Explore whether changes to work made by participants over a 3‐month period impacted their job satisfaction and/or mental wellbeing.

## 2 Method

### 2.1 Research Design/Context

This article reports quantitative findings from a longitudinal study, that was part of a larger mixed‐methods project. Data was collected in 2018, using an online questionnaire that was available at two time points 3 months apart.

### 2.2 Ethics

Ethical approval was obtained from The Business, Law, and Social Sciences Faculty Research Committee at Birmingham City University and all respondents provided written and informed consent to participate.

### 2.3 Participants

The theoretical population being studied consisted of all SLTs practising clinically in the UK, at the time that the study took place. *Practising clinically* was operationalised as holding an active caseload. Inclusion criteria were therefore (a) qualified SLTs working in the UK, who were (b) practising clinically.

### 2.4 Recruitment

To encourage candid responses about work experiences, potential participants were contacted independent of their employing organisations. Failure to procure a membership list of the profession prohibited random sampling, meaning that selective sampling combined with snowballing was used. This form of nonprobability sampling is potentially susceptible to bias, which may threaten the generalisability of findings. However, it is appropriate when an exercise aims to gather information to describe a specific group, and a population listing (or in this case membership list) is not available. In January 2018, an article was published in ‘Bulletin’, the professional magazine published by the Royal College of Speech and Language Therapists (RCSLT, the professional body for SLTs in the UK), describing the study and providing contact details for the primary researcher. Following this, respondents (*n* = 68) were emailed a link to the questionnaire. In addition, 56 of the 116 Clinical Excellence Networks (CENs) in the UK were emailed with information about the study and a link to the questionnaire (60 CENs with National Health Service email addresses were not contacted, as NHS Health Research Authority approval was not sought for this study). Following this, ‘passive’ recruitment was used, through publication of the link to the questionnaire via the following web‐based channels: the RCSLT e‐Research newsletter; the online Association of Speech and Language Therapists in Independent Practice^1^ forum (available to all ASLTIP members) and the Independent Practitioners in Speech and Language Therapy^2^ Yahoo Group. Finally, social media was used. A direct message was sent via X (then Twitter) to UK‐based SLTs who followed the primary researcher. A general public tweet was not sent, as this risked the questionnaire being available to non‐SLTs. Seven weeks after the survey was initially made available, further funding was procured, and an advertisement for the study was published in the RCSLT Bulletin in June 2018. In total, the initial survey was available to SLTs from 16 April 2018 to 18 July 2018 for the first phase (P1). In September 2018, the second questionnaire was emailed to the 480 participants who had agreed, at P1, to complete a follow‐up survey. Of these, three returned automatic messages stating that the person in question was no longer in post. A further 22 returned ‘undeliverable’ messages. Due to the potential that emails were rejected owing to being part of a mass mailing, individual emails were sent to these addresses, and seven participants were reached. This meant that of the 480 potential participants, 462 (96.3%) emails were delivered. By the beginning of October 2018, 235 participants had responded. A reminder email was sent to the remaining 228 people who had provided an email address but who had not yet completed the questionnaire. On 14 October, 327 (70.8%) responses had been received. The survey was closed at this time, having been made available for a month for phase two (P2).

### 2.5 Sample Size

**FIGURE 2:**
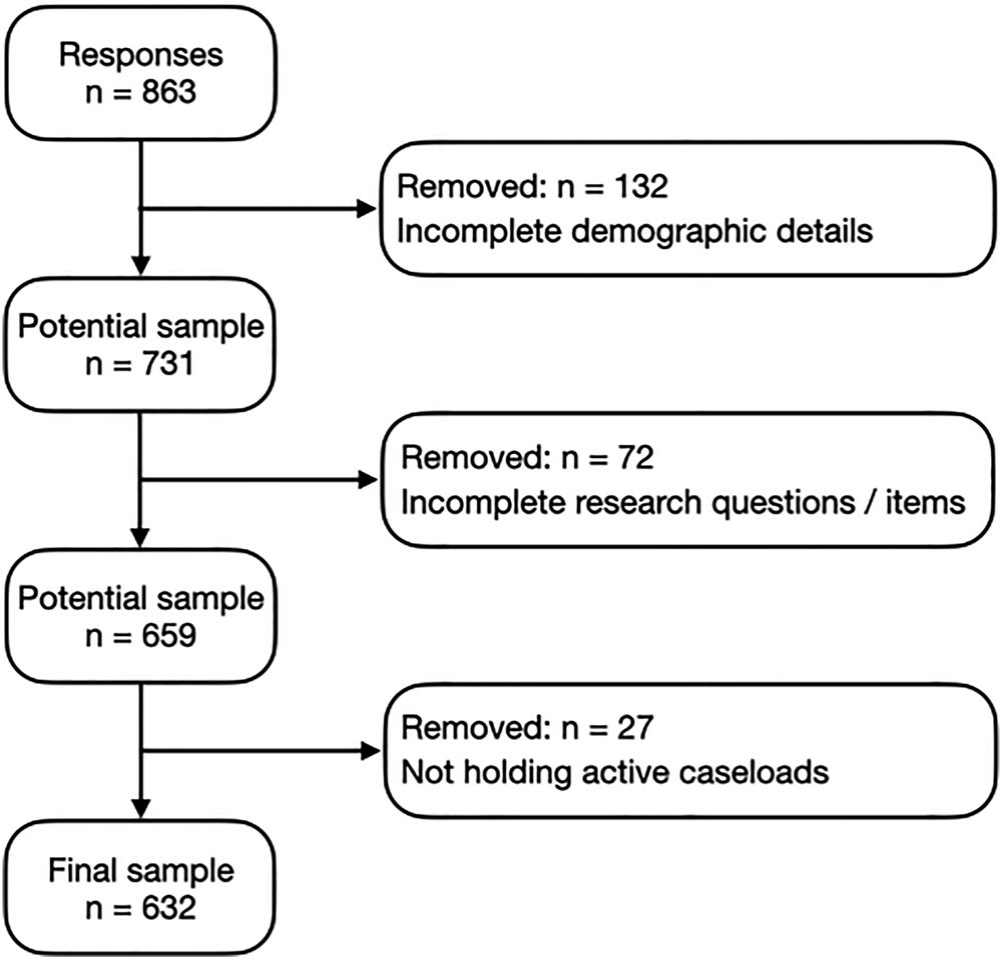
Flowchart of the inclusion process for final sample at P1.

**FIGURE 3:**
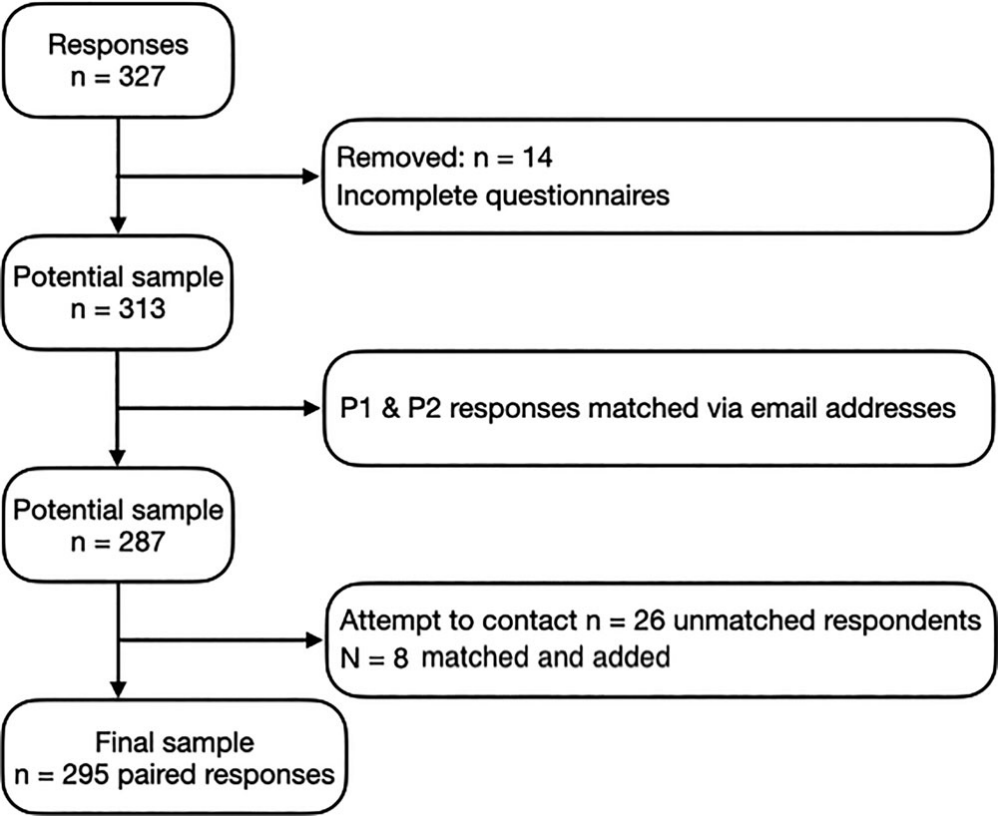
Flowchart of the inclusion process for participants at P2.

To determine the sample size needed to facilitate any relationship between the variables being investigated, a power analysis was performed, using G*Power 3.1.9.2 (Faul et al. 2007). The current study aimed to use the General Health Questionnaire (GHQ) to measure wellbeing. Therefore, previous studies utilising the GHQ were used to determine an estimated effect size. The calculation employed a significance level of *p* = 0.05, minimum power of 0.80 and a small possible effect size (*r* ^2^ = 0.03), and indicated that a sample size of 607 was required. The original online survey received 863 responses from UK‐based SLTs, and Figure 2 shows the flowchart for the inclusion process of participants at P1. The process for inclusion of participants at P2 is depicted in Figure 3.

### 2.6 Procedures

#### 2.6.1 Materials

A questionnaire for self‐completion by participants was designed by the researchers for interactive online use. It was piloted in January 2018 and issues that were identified (e.g., lack of clarity for some questions, too long taken to complete the questionnaire) were addressed.

The questionnaire at P1 included mostly closed‐ended questions and consisted of five sections:

- participant demographics,
- working profiles, for example, full time/part time, tenure in current job,
- job characteristics and psychosocial work environments, that is, caseload size, amount of control over service delivery, professional support received,
- dispositional traits, for example, self‐esteem, optimism (these results, and the mediating effect that they had on the relationship between work and wellbeing, will be reported elsewhere),
- wellbeing, for example, job satisfaction, anxiety, depression.

The final Section (v) that investigated wellbeing contained the following published scales:

- The Speech‐Language Pathologist Stress Inventory (SLPSI, Fimian et al. 1991). The SLPSI has four subscales: ‘Time and Workload Management’ (TWM), ‘Bureaucratic Restrictions’ (BR), ‘Instructional Limitations’ (IL) and ‘Lack of Professional Support’ (LPS). The possible range of scores on the SLPSI is from 1 (*never*) to 5 (*always*).
- The 10‐item Generic Job Satisfaction Scale (GJSS, Macdonald and MacIntyre 1997). Job satisfaction scores on the GJSS are categorised from 10 (*very low*) to 50 (*very high*).
- The General Health Questionnaire – 28 (GHQ‐28, Goldberg 1972). GHQ‐28 scores, measuring general psychosocial wellbeing, are calculated using a binary method (with the two least symptomatic answers scoring 0 and the two most symptomatic answers scoring 1). The GHQ‐28 classifies any total score meeting the threshold value of 4 as achieving ‘caseness’ (Jackson 2007). This ‘caseness’ is an indication of psychological distress. The binary scores can be further classified into three bands (Nolan and Ryan 2008): no stress, mild stress (likely to resolve with intervention) and severe stress (unlikely to resolve without intervention).

Reliability of the outcome scales was assessed for this study and is reported in the Results section.

At P2, only the GJSS and GHQ‐28 were retained, and two questions were added:

- *Are you still in the same job that you were in when you answered the first questionnaire between April and July 2018?*
- *Have you personally made any positive personal changes that you believe have altered your well‐being? (e.g., no longer taking work home/started an exercise programme* */other)*.

### 2.7 Missing Data

The protocol for dealing with missing data required the replacement of missing values with the mean:

1. GJSS: the mean score for the scale, using completed items, was computed and used to replace missing responses. The same procedure was used with those who had selected ‘not applicable’ on any item.
2. SLPSI and GHQ‐28: the mean score for each subscale, using completed items, was calculated. Missing values in particular subscales were then replaced with the mean for that subscale.

For some variables, for example, date of birth or year qualified, using a mean to replace a missing value was not appropriate. If values were missing for these variables, they were left blank, reducing the number of participants included for the analysis of these variables.

### 2.8 Data Analysis

Frequency counts, mean scores and standard deviations were used to establish distribution and central tendency for all variables.

For all of the analysis, constructs were operationalised as follows:

- Predictor/contributor variables: Job types were classified according to the JDCS categories, described in the introduction.
- Outcome variables: Occupational wellbeing was operationalised as job satisfaction and measured by the GJSS; and general wellbeing was measured by the GHQ‐28.

Table 1 presents the scales used, how these were mapped to the JDCS model, scoring, levels indicated by scores and classification of these scores.

**TABLE 1:** Scales, theory/models, scores, levels and classifications used for inferential analysis.

	Underlying theory/theoretical model	Instrument	Score	Classification
Contributors to job ‘type’		Speech‐Language Pathologist Stress Inventory subscales		
	Demand	Time and Workload Management	1.00–3.00	Low demand
			3.01–5.00	High demand
	Control	Professional Autonomy^a^ (Bureaucratic Restrictions + Instructional Limitations)^b^	1.00–3.00	High control
			3.01–5.00	Low control
	Support	Lack of professional support^b^	1.00–3.00	High support
			3.01–5.00	Low support
		Job component combinations		
Predictor variable: Job ‘type’	JDCS model (Johnson and Hall 1988; Karasek 1979)	High support	High demand – high control	Active collective
			High demand – low control	High‐strain collective
			Low demand – high control	Low‐strain collective
			Low demand – low control	Passive collective
		Low support	High demand – high control	Active isolated
			High demand – low control	High‐strain isolated (IsoStrain)
			Low demand – high control	Low‐strain isolated
			Low demand – low control	Passive isolated
Outcome variable: Job satisfaction		Generic Job Satisfaction Scale	10–26	Very low
			27–31	Low
			32–38	Average
			39‐41	High
			42–50	Very high
Outcome variable: Wellbeing		General Health Questionnaire‐28	Binary total: 0–3	No distress
			Binary total: 4–7	Mild distress
			Binary total: 8	Severe distress
			Scale total > 23	Risk of psychiatric disorder

^a^ Bureaucratic restrictions and instructional limitations were grouped to create one scale, Professional Autonomy (PA), which represented ‘control’.

^b^ Bureaucratic restrictions, instructional limitations and lack of professional support consist of negatively worded items and were therefore reverse‐scored for classification purposes.

Inferential analysis was as follows:

- The wellbeing of participants across the different JDCS job types was compared, using a one‐way multivariate analysis of variance (MANOVA), follow‐up univariate analyses of variance tests (ANOVAs) and Games‐Howell post‐hoc tests.
- Changes in wellbeing outcomes across time were first compared using *t* tests.
- A three‐way mixed MANOVA and follow‐up *t* tests were then conducted to explore the effects of a change of job and/or positive personal changes on job satisfaction (GJSS scores) and general wellbeing (GHQ‐28 scores) over a 3‐month period.

## 3 Results

### 3.1 Participants

Due to the multifaceted method of participant recruitment, it is not possible to report a response rate. The 632 SLTs in this sample represented approximately 4% of the 15 932 SLTs that were registered to practice in the UK in 2017, the latest figures available when the survey took place in 2018.

### 3.2 Demographics

Of the SLTs in this sample, 98% were women, which was consistent with the population demographics of UK SLTs at the time. Ninety‐five percent were white, and the average age of participants was 38.7 years. Of the sample, 91% were working in England and 85% were organisationally employed. Seventy‐six percent were employed in some capacity by the NHS (67% worked only in the NHS, and 9% worked in the NHS part‐time and elsewhere part‐time). The NHS provides services to schools and nurseries, and the majority of the sample (45%) worked in the education sector. Three fifths of participants worked full‐time, and 42% earned between £20 000 and £29 000. The bulk of participants (85%) worked extra unpaid hours in addition to their contracted hours. The mean caseload size was 57, with those working in schools carrying the largest caseloads (*M* = 74 for mainstream schools, *M* = 87 for secondary special schools). On average, 38% of an SLT's time was spent delivering face‐to‐face intervention. Most (93%) responded to emails throughout the day, and 40% read and sent emails outside of working hours. Most participants reported having physically demanding jobs, with 80% being required to engage in manual handling and/or carrying/moving heavy equipment.

### 3.3 Job Satisfaction and General Wellbeing Levels

Three scales were used to describe SLT wellbeing:

i. SLPSI: the mean ‘Total Stress Score’ for the current sample was 2.7 (SD = 0.50), indicating ‘moderate’ levels of stress (Fimian et al. 1991).
ii. GJSS: the mean score for the sample was 36.31 (SD = 6.54), that is, within the ‘average’ band for job satisfaction.
iii. GHQ‐28: 15 participants did not complete the GHQ‐28 and were excluded from further analysis that utilised this scale. Of the 617 participants who completed the GHQ‐28, 53.3% (*n* = 329) had binary scores of 4 or more, indicating some level of distress.

Table 2 presents the distribution of scores for the three scales.

**TABLE 2:** The distribution of scores across SLPSI (*n* = 632), GJSS (*n* = 632) and GHQ‐28 (*n* = 617) bands.

Banding	*n* (%)
SLPSI stress levels	
1.00–1.99	50 (8)
2.00–2.99	386 (61)
3.00–3.99	195 (30.8)
4.00–4.99	1 (0.2)
5.00	0 (0)
GJSS	
10–26	50 (8)
27–31	88 (14)
32–38	240 (38)
39–41	120 (19)
42–50	133 (21)
GHQ‐28	
≤3	288 (46.7)
4–6	91 (14.7)
≥7	238 (38.6)

On the SLPSI, participants scored worst on the TWM subscale. The three items where participants scored the highest were having too much to do, too much administrative work, and being overcommitted.

On the GJSS, 85% (*n* = 540) of participants agreed or strongly agreed that they had good relationships with their supervisors. In contrast, only 40% (*n* = 253) felt that management was concerned about them.

On the GHQ‐28, participants scored worst on the subscale measuring their ability to function well on a social level (i.e., the social dysfunction subscale). The three items where SLTs scored highest on the GHQ‐28 were feeling run down and ‘out of sorts’, not feeling well and in good health, and taking longer than normal to do things.

### 3.4 Reliability

Reliability across all subscales was acceptable (i.e., greater than 0.6), with scale totals demonstrating excellent reliability.

The Cronbach's alpha scores, mean scores and standard deviations for each of the scales used are presented in Table 3.

**TABLE 3:** Cronbach's alpha and mean scores for the SLPSI, GHQ‐28 and GJSS.

SLPSI subscales	Cronbach's alpha^a^	Mean (SD)
Time and workload management^b^	0.894	3.50 (0.77)
Bureaucratic restrictions^b^	0.847	2.79 (0.83)
Instructional limitations^b^	0.692	2.69 (0.55)
Lack of professional support^b^	0.792	2.62 (0.61)
Biobehavioural manifestations^c^	0.680	1.94 (0.64)
Emotional‐fatigue manifestations^c^	0.816	2.55 (0.58)
SLPSI total	**0.933**	**2.71 (0.50)**

GHQ‐28 subscales	Cronbach's alpha^a^	Mean (SD)
Somatic symptoms	0.857	7.08 (4.4)
Anxiety	0.903	7.13 (5.0)
Social dysfunction	0.813	8.08 (2.6)
Depression	0.881	1.58 (2.9)
GHQ‐28 Likert total	**0.932**	**23.85 (12.1)**
GHQ‐28 Binary total	**0.915**	**5.90 (6.0)**

GJSS Items	Cronbach's alpha^a^	Mean (SD)
I get along with my supervisors		4.26 (0.76)
I feel close to the people at work		3.93 (0.92)
I feel good about my job		3.88 (0.96)
I feel good about working for this service/practice/company/myself		3.84 (1.0)
I feel secure about my job		3.76 (1.0)
I receive recognition for a job well done		3.65 (.95)
On the whole, I feel work is good for my health		3.49 (1.1)
All my talents and skills are used at work		3.34 (1.1)
My wages are good		3.08 (1.1)
I believe management is concerned about me		3.06 (1.24)
GJSS total	**0.838**	**36.31 (6.54)**

^a^ Cronbach's alpha score > 0.6 is acceptable.

^b^ SLPSI contributor subscales.

^c^ The mean score for all 16 SLPSI *Outcomes of Stress* items (i.e., items from both the Bio‐behavioural Manifestations and the Emotional‐Fatigue Manifestations subscales) was 2.32 (SD = 0.54).

**TABLE 4:** GJSS and GHQ‐28 mean scores for the JDCS model groups (*n* = 632).

Job type (number of participants, percentage of sample)	GJSS *M* (SD)	GJSS satisfaction score	GHQ‐28 *M* (SD)	Risk of psychiatric disorder?
IsoStrain (*n* = 105, 17%)	29.1 (5.9)	Low	32.5 (13.7)	Yes
Passive isolated (*n* = 5, 0.8%)	29.6 (1.9)	Low	23.2 (10.4)	Yes
High‐strain collective (*n* = 101, 16%)	33.6 (5.2)	Average	27.9 (11.6)	Yes
Active isolated (*n* = 31, 5%)	33.2 (5.3)	Average	30.2 (11.3)	Yes
Passive collective (*n* = 8, 1.2%)	32.5 (6.6)	High	22.8 (10.7)	Borderline
Active collective (*n* = 222, 35%)	38.5 (4.7)	High	22.9 (10.7)	Borderline
Low‐strain isolated (*n* = 7, 1%)	34.9 (3.4)	Average	21.4 (10.5)	No
Low‐strain collective (*n* = 153, 24%)	40.2 (5.3)	High	16.7 (7.7)	No

### 3.5 Comparison of Job Satisfaction and General Wellbeing for Different Job Types

The JDCS classification of the sample and the scores for all eight groups are presented in Table 4. Those in IsoStrain (high demand, low support and little control) jobs had the lowest job satisfaction scores and worst wellbeing scores.

**FIGURE 4:**
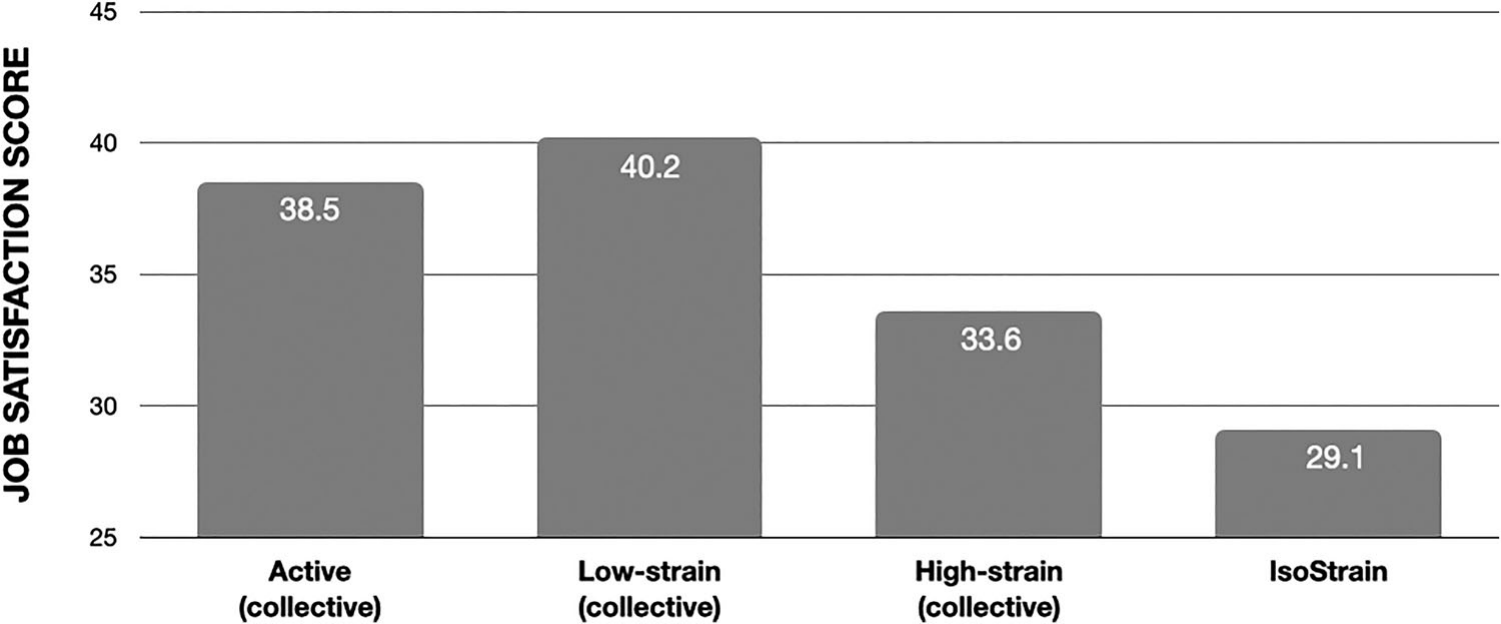
Generic Job Satisfaction Scale (GJSS) scores for the four job types retained for analysis (*n* = 581).

**FIGURE 5:**
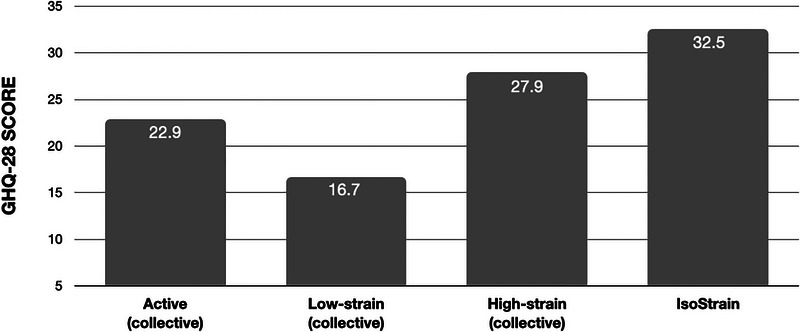
General Health Questionnaire‐28 (GHQ‐28) scores for the four job types retained for analysis (*n* = 581).

A one‐way MANOVA was conducted to determine the relationship between job type (according to the JDCS model) and wellbeing. Of the eight possible job classifications that the JDCS model includes (displayed in Table 4), four groups represented only 8% of the sample (Active Isolated, 5%; Passive Collective, 1.2%; Passive Isolated 0.8%; Low Strain Isolated, 1%). Due to the very small numbers in these four groups, they were not included in further analysis. The remaining four groups compared in the final analysis were the Active Collective, Low‐strain Collective, High‐Strain Collective and IsoStrain groups, with GJSS scores and GHQ‐28 scores depicted in Figures 4 and 5.

The differences between the four groups on the combined dependent variables was statistically significant, *F* (6,126) = 52.016, *p* < 0.001; Pillai's Trace = 0.434; partial *η*2 = 0.217. Follow‐up univariate ANOVAs showed that both job satisfaction scores, *F* (3,563) = 116.507, *p* < 0.001, partial *η*2 = 0.383, and general biopsychosocial well‐being scores, *F* (3,563) = 49.932, *p* < 0.001, partial *η*2 = 0.210, were statistically significantly different between the SLTs in the different groups. Partial eta‐squared values indicated a large effect size for all differences.

Games–Howell post‐hoc tests showed that the difference in job satisfaction scores was statistically significant between all groups.

For general wellbeing, Games–Howell post‐hoc tests also showed that scores were statistically significantly different between all groups apart from the High‐Strain Collective and IsoStrain groups, which narrowly failed to reach significance (*p* = 0.059). Scores are shown in Table 5.

**TABLE 5:** Difference in GJSS and GHQ‐28 scores for the four retained job types (*n* = 581).

GJSS scores					
				95% Confidence interval (CI)	
JDCS Group (a)	JDCS Group (b)	Mean difference between (a) and (b)	*p*	Lower CI	Higher CI
Active collective	Low‐strain collective	−1.69	**0.013**	−3.13	−0.26
	High‐strain collective	4.94	**<0.001**	3.47	6.42
	Isostrain	9.43	**<0.001**	7.67	11.18
Low‐strain collective	High‐strain collective	6.63	**<0.001**	4.95	8.32
	Isostrain	11.12	**<0.001**	9.19	13.05
High‐strain collective	Isostrain	4.49	**<0.001**	2.52	6.45

GHQ‐28 scores					
				95% Confidence interval (CI)	
JDCS Group (a)	JDCS Group (b)	Mean difference between (a) and (b)	*p*	Lower CI	Higher CI
Active collective	Low‐strain collective	6.20	**<0.001**	3.74	8.65
	High‐strain collective	−4.91	**0.003**	−8.54	−1.29
	Isostrain	−9.55	**<0.001**	−13.56	−5.53
Low‐strain collective	High‐strain collective	−11.11	**<0.001**	−14.69	−7.53
	Isostrain	−15.75	**<0.001**	−19.72	−11.79
High‐strain collective	Isostrain	−4.64	0.059	−9.40	0.12

### 3.6 Consistency of Job Satisfaction and Wellbeing Over Time

Job satisfaction and general wellbeing levels were then compared over a 3‐month period. The second survey (P2) yielded 295 paired responses. Firstly, changes in job satisfaction and general wellbeing across the whole sample were compared. Table 6 reports mean scores.

**TABLE 6:** GJSS and GHQ‐28 mean scores for P1 and P2.

GJSS, *n* = 286 *M* (SD)		GHQ‐28, *n* = 283 *M* (SD)	
P1	P2	P1	P2
37.07 (6.40)	37.59 (6.14)	21.52 (10.20)	21.98 (10.10)

A paired samples *t* test revealed that the increase in GJSS score across time was statistically significant, *t* (285) = −2.04, *p* = 0.04. However, the increase in the GHQ‐28 score across time was not statistically significant, *t* (282) = −0.89, *p* = 0.37.

### 3.7 Effects of Changes Made on Wellbeing

A three‐way mixed MANOVA was conducted to explore the effects of making positive personal changes (e.g., no longer taking work home, starting an exercise programme) and/or changing jobs, on job satisfaction (GJSS scores) and general wellbeing (GHQ‐28 scores). The three independent variables were therefore time (P1 and P2), job (same or not) and positive personal change (or lack thereof). The two dependent variables were the GJSS and GHQ‐28 scores.

#### 3.7.1 Job Satisfaction

Changes in job satisfaction were analysed for 292 respondents (three participants who had left their jobs were no longer working). The three‐way interaction between time, job changes and positive personal changes was not statistically significant for job satisfaction, *F* (1,284) = 0.532, *p* = 0.47, partial *η*2 = 0.002. There was also no statistically significant two‐way interaction between time and positive personal changes on GJSS scores, *F* (1,284) = 0.478. *p* = 0.49, partial *η*2 = 0.002. However, there was a statistically significant two‐way interaction between time and job changes on GJSS scores, *F* (1,284) = 8.07, *p* = 0.005, partial *η*2 = 0.028. The difference in GJSS scores between P1 and P2 for the two groups (‘remained in job’ vs. ‘left job’) was therefore explored in more detail. Only seventeen participants had changed jobs between P1 and P2, meaning the sample size for this group was small. Table 7 reports the mean job satisfaction scores for the two groups at P1 and P2.

**TABLE 7:** Mean job satisfaction scores at P1 and P2 for those who changed jobs and those who remained in their jobs.

	Mean GJSS score (SD)^a^	
	P1	P2
Same job (*n* = 275)	37.16 (6.52)	37.50 (6.41)
Different job (*n* = 17)	33.06 (6.67)	37.88 (5.77)

^a^ Scores between 32 and 38 denotes ‘average’ job satisfaction.

To explore the difference in job satisfaction between the two groups at P1 and then at P2, *t* tests were used. At P1, SLTs who later remained in their jobs had higher GJSS scores (*M* = 37.16, SD = 6.52) than those who later left their jobs (*M* = 33.06, SD = 6.67), and this difference was statistically significant, *t* (292) = 3.285, *p* = 0.001. At P2, there was an improvement in GJSS scores for both the SLTs who had left their previous jobs (*M* = 37.88, SD = 5.77) and those who remained (*M* = 37.50, SD = 6.41) but the difference in GJSS between the two groups at P2 was not statistically significant, *t* (290) = −0.241, *p* = 0.81.

**FIGURE 6:**
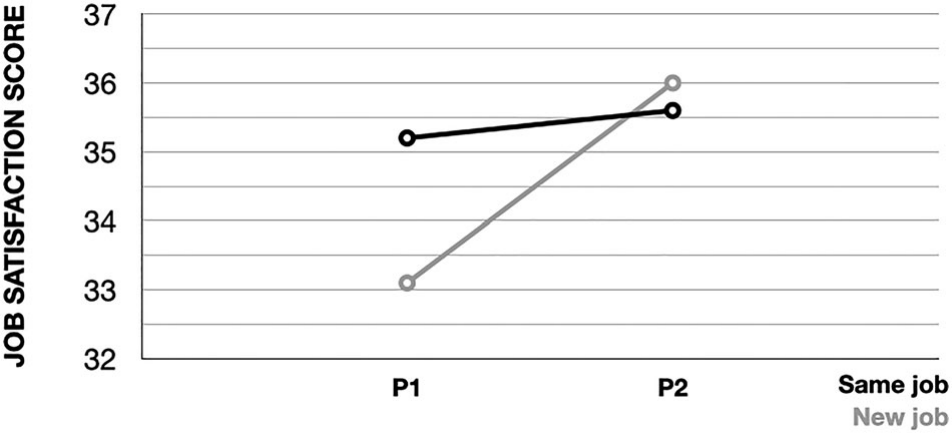
Changes in job satisfaction over time, for speech and language therapists (SLTs) who remain in their jobs and those who leave and start new jobs.

Finally, the difference in job satisfaction for each group across time (i.e., between P1 and P2) was explored. For the seventeen respondents who had changed jobs, there was a statistically significant increase in job satisfaction, *t* = −3.225, *p* < 0.01. For those who were in the same job at the two time points (*n* = 275), there was not a significant change, *t* (274) = −1.09, *p* = 0.28. Figure 6 illustrates the changes in GJSS scores for the two groups over time, with the graph focussing specifically on the ‘average’ band of the GJSS.

#### 3.7.2 General Wellbeing

There was no statistically significant three‐way interaction between time, job changes and positive personal changes for general wellbeing, *F* (1,284) = 0.058, *p* = 0.81, partial *η*2 = 0.000. There were also no statistically significant interactions between time and positive personal changes on GHQ‐28 scores, *F* (1,284) = 1.04, *p* = 0.31, partial *η*2 = 0.004, or between time and job changes on GHQ‐28 scores, *F* (1,284) = 1.882, *p* = 0.17, *η*2 = 0.007.

## 4 Discussion

To our knowledge, this is the first study to investigate the general wellbeing of SLTs, beyond job satisfaction. Data was collected in 2018, before the onset of COVID‐19, and therefore describes the ‘typical’ working life of SLTs, and their wellbeing associated with this work; without the confounding experiences of practicing during a pandemic. The study compared data collected from SLTs across the UK at two time points; Phase One (April to July 2018) and Phase Two (September to October 2018).

### 4.1 Levels of General Wellbeing and Consistency Over Time

The majority of SLTs in the sample achieved ‘caseness’ on the GHQ‐28, meaning that should they present at their GP; their symptoms (e.g., levels of anxiety) would attract further attention (Jackson 2007). In addition, a significant number were classified as having severe distress, that is, symptoms that were unlikely to resolve without intervention. Scores were stable over a 3‐month period, indicating that responses were unlikely to be the result of transient mood.

The GHQ‐28 has not been used with SLTs before, and results can therefore not be compared to other SLTs. It has, however, been employed in numerous studies investigating general wellbeing in other occupational groups and general populations. The current cohort's scores were worse than those that have previously been achieved by health workers in the UK (Wall et al. 1997), but better than those achieved by South African nurses (Khamisa et al. 2017). Considering Karasek's (1979) theory regarding the relationship between employment conditions and wellbeing, a cautious interpretation of these differences may be that work environments differ in these assorted professions, or in these varied locations, resulting in dissimilar levels of general wellbeing.

### 4.2 Levels of Job Satisfaction and Consistency Over Time

Research in the USA has shown some SLTs to have high job satisfaction (Hutchins et al. 2010). The current UK‐based study found that the majority of the survey participants had average/high job satisfaction, providing some corroboration of existing information. However, a significant minority of participants surveyed reported low or very low job satisfaction.

The longitudinal findings of the survey revealed a small, but significant increase in job satisfaction over 3 months.

#### 4.2.1 The Effect of Personal Changes

Making positive personal changes between the two time points did not improve job satisfaction. This finding is consistent with that of Fleming (2024), who questioned the efficacy of common wellbeing interventions (e.g., wellbeing apps, mindfulness programmes and relaxation training), reporting no reliable difference in mental wellbeing between workers who had received intervention and those who had not. It is, however, in contrast with previous research that does demonstrate that personal wellbeing strategies improve health in the workplace (Puerto Valencia et al. 2019). This difference could be because the current study required participants to report whether they had made any ‘positive personal changes’ between the two questionnaires but did not control for what those changes were, how they were implemented, or whether there were confounding influences, therefore making comparison problematic.

#### 4.2.2 The Effect of Changing Jobs

Although the subgroup of SLTs who left their jobs was small, they had significantly lower job satisfaction at the time of the first survey than those who did not leave. Interpreted cautiously, this could indicate that those who are unhappiest choose to resign from their roles. Changing jobs resulted in an improvement in job satisfaction. Examining whether this improvement is maintained or whether it was a temporary spike in satisfaction was not within the scope of this study.

### 4.3 The Relationship Between Job Types and Wellbeing

The JDCS model (Johnson and Hall 1988; Karasek 1979) enabled exploration of the combined effect of the psychosocial elements of demand, control and support within the work environment on the wellbeing of SLTs. The model was applied to the data collected from the survey, and job types were identified. Of this sample, almost one fifth were in IsoStrain jobs. Consistent with several research studies (e.g., Nieuwenhuijsen et al. 2010) participants who experienced high demand, low control and low support were more likely to experience case‐level psychological distress and low job satisfaction. Conversely, those SLTs in Active Collective/Low‐strain Collective jobs had the best general wellbeing and the highest job satisfaction. This study therefore provides novel information about SLT jobs and how these are associated with wellbeing, as well as further corroboration for previous findings linking jobs with high demands and low control to negative psychological wellbeing (van Doorn et al. 2016), and low levels of job satisfaction (Häusser et al. 2010).

### 4.4 Clinical Implications

Healthy SLTs are more likely to stay in their jobs (Loan‐Clarke et al. 2009) and to have the personal resources to support their service users effectively. The first step in supporting SLT wellbeing is to ensure that there is a clear understanding of the psychosocial composition of their jobs. The focus of this study has been broad, without the scope to assess individual workplaces. Leka et al. (2015) argue that psychosocial risks are embedded within work organisation, although a false distinction between the two is sometimes made. This study has revealed that a combination of potential workplace features, including demands, control (autonomy) and support, are associated with SLT job satisfaction, suggesting employers consider the overall psychosocial design of SLT jobs, instead of viewing occupational factors individually. The JDCS model has proved a suitable framework for describing SLT jobs, and it could be used in the future to gain detailed knowledge about different elements that constitute particular SLT jobs. A useful resource for employers in the UK is the Health and Safety Executive Management Standards Indicator Tool and workbook, a step‐by‐step risk assessment and intervention approach (Health and Safety Executive Standards, n.d.). Risk is likely to be different in different settings, and depending on the employer. It is essential that the voices of SLTs who are primarily clinical are heard and used to inform this appraisal; collaboration between managers and clinicians is necessary for success in this endeavour. Working parties could facilitate this appraisal, which should include details of the demands (physical, emotional, psychological and in terms of workload), control (e.g., levels of autonomy regarding service delivery) and support (supervision and informal support) that characterise a job. This work will potentially be difficult, given the prevailing culture within healthcare, which places responsibility for wellbeing on the individual by encouraging self‐reliance and coping (Harvey et al. 2009). However, clear knowledge of the psychosocial elements of SLT jobs is the starting point for change, and once this knowledge is in place, areas that require development can be addressed.

### 4.5 Strengths and Limitations

This research was the first of its kind to directly investigate the occupational as well as general wellbeing of SLTs practising clinically in the UK, to use a theoretical model to frame the research and to focus on wellbeing per se. With 632 participants at P1 and 295 paired responses at the follow‐up, this is also the largest study of its kind in the UK. Participants were recruited from across the country and included paediatric SLTs and those working with adults, as well as employed and self‐employed SLTs, possibly supporting the generalisation of findings. A further strength of this study was its longitudinal element, as repeated measurement can go some way to ameliorating the effects of common method variance, including mood and context (Siegrist et al. 2004).

A limitation of the study was that self‐report was used, which could have resulted in participant response bias, with either the happiest or the most stressed SLTs not being represented. Although unequivocally identifying whether the survey results were affected by response bias is not straightforward, the questionnaire was carefully designed and piloted in an attempt to minimise this. A further limitation was that the survey instrument used was published online. Although self‐administered survey modes may reduce the impact of social desirability, the online survey could have resulted in selection bias, resulting in an unrepresentative sample. At P2, the number of participants who had changed jobs was small, meaning that cautious interpretation of findings is prudent. Finally, the questionnaire at P2 did not include questions about job factors, meaning that a cause‐effect relationship between work and wellbeing could not be established.

## 5 Conclusion

Results of this study indicate that, before the onset of COVID‐19, just over half of SLTs working clinically in the UK were at risk of being psychologically vulnerable to anxiety, depression, experiencing somatic symptoms of stress and social dysfunction. This is a worrying finding, suggesting that there were psychological health issues in the workforce before the COVID‐19 pandemic commenced, and having possible implications for SLT career longevity.

More recent studies have focussed on the effects of working during COVID‐19 on the wellbeing and job satisfaction of health workers (e.g., Gilleen et al. 2022) and SLTs more specifically (Farquharson et al. 2021). Although long‐term effects of the psychological impact of working as an SLT during the pandemic are currently unknown, there is a danger of presuming that SLTs enjoyed high levels of mental health and job satisfaction pre‐COVID‐19. It is arguably important to establish wellbeing indicator benchmarks associated with typical jobs for comparison purposes and to guard against return to a ‘normal’ that might not be supportive of worker wellbeing. Currently, postpandemic research continues to report burnout and extreme emotional exhaustion in SLTs (Coleman et al. 2024), suggesting poor wellbeing continues to be an area requiring attention.

Although some stakeholders consider psychosocial risks difficult to address in a preventative fashion, focussing on opportunities and resources is necessary to mitigate risk (Leka et al. 2015). Findings of this study suggest that SLTs with the lowest job satisfaction leave their jobs. There is therefore a need for holistic appraisal of SLT job design to ensure that workplaces provide the environments necessary for promoting manageable demands, effective support and genuine autonomy. This appraisal must include input from front‐line practitioners and can determine whether and how the design of jobs requires addressing. Finally, this research is a first step in the attempt to understand and support the wellbeing of SLTs associated with their typical jobs. It is a snapshot of their lives and as such, given the findings, it is the ethical responsibility of researchers to continue to study this neglected area to assist in attempts to ensure that psychosocial risks are managed, and hazards are remedied.

## Ethics Statement

Ethical approval was obtained from The Business, Law and Social Sciences Faculty Research Committee at Birmingham City University.

## Consent

All respondents provided written and informed consent to participate.

## Conflicts of Interest

The authors declare no conflicts of interest.

## Data Availability

The data that support the findings of this study are available from the corresponding author upon reasonable request. The data are not publicly available due to privacy or ethical restrictions.

## Endnotes

1 The Association of Speech and Language Therapists in Independent Practice (ASLTIP) is an organisation to which many independent SLTs in the UK voluntarily belong.

2 Independent Practitioners in Speech and Language Therapy (IPSLT) is an online support group for any SLTs in independent practice in the UK.
